# On properties of geodesic semilocal E-preinvex functions

**DOI:** 10.1186/s13660-018-1944-z

**Published:** 2018-12-20

**Authors:** Adem Kılıçman, Wedad Saleh

**Affiliations:** 1 Department of Mathematics and Institute for Mathematical Research, University Putra Malaysia, Serdang, Malaysia; 2 Department of Mathematics, Taibah University, Al-Medina, Saudi Arabia

**Keywords:** Generalized convexity, Riemannian geometry, Duality

## Abstract

The authors define a class of functions on Riemannian manifolds, which are called geodesic semilocal E-preinvex functions, as a generalization of geodesic semilocal E-convex and geodesic semi E-preinvex functions, and some of its properties are established. Furthermore, a nonlinear fractional multiobjective programming is considered, where the functions involved are geodesic E-*η*-semidifferentiability, sufficient optimality conditions are obtained. A dual is formulated and duality results are proved by using concepts of geodesic semilocal E-preinvex functions, geodesic pseudo-semilocal E-preinvex functions, and geodesic quasi-semilocal E-preinvex functions.

## Introduction

Convexity and generalized convexity play a significant role in many fields, for example, in biological system, economy, optimization, and so on [1–5].

Generalized convex functions, labeled as semilocal convex functions, were introduced by Ewing [6] by using more general semilocal preinvexity and *η*-semidifferentiability. After that optimality conditions for weak vector minima were given [7]. Also, optimality conditions and duality results for a nonlinear fractional involving *η*-semidifferentiability were established [8].

Furthermore, some optimality conditions and duality results for semilocal E-convex programming were established [9]. E-convexity was extended to E-preinvexity [10]. Recently, semilocal E-preinvexity (SLEP) and some of its applications were introduced [11–13].

Generalized convex functions in manifolds, such as Riemannian manifolds, were studied by many authors; see [14–17]. Udrist [18] and Rapcsak [19] considered a generalization of convexity called geodesic convexity. In this setting, the linear space is replaced by a Riemannian manifold and the line segment by a geodesic one. In 2012, geodesic E-convex (GEC) sets and geodesic E-convex (GEC) functions on Riemannian manifolds were studied [20]. Moreover, geodesic semi E-convex (GsEC) functions were introduced [21]. Recently, geodesic strongly E-convex (GSEC) functions were introduced and some of their properties were discussed [22].

## Geodesic semilocal E-preinvexity

Assume that ℵ is a complete *n*-dimensional Riemannian manifold with Riemannian connection ▽. Let $\kappa _{1}, \kappa _{2} \in \aleph $ and $\gamma \colon [0,1]\longrightarrow \aleph $ be a geodesic joining the points $\kappa _{1} $ and $\kappa _{2} $, which means that $\gamma _{\kappa _{1},\kappa _{2}}(0)= \kappa _{2}$ and $\gamma _{\kappa _{1},\kappa _{2}}(1)=\kappa _{1} $.

### Definition 2.1

A nonempty set $B \subset \aleph $ is said to be

1. a geodesic E-invex (GEI) with respect to *η* if there is exactly one geodesic $\gamma _{E(\kappa _{1}), E(\kappa _{2})}: [0,1 ] \longrightarrow \aleph $ such that
  $$\begin{aligned} \gamma _{E(\kappa _{1}), E(\kappa _{2})}(0)=E(\kappa _{2}), \qquad \acute{\gamma }_{E(\kappa _{1}), E(\kappa _{2})}=\eta \bigl(E(\kappa _{1}),E( \kappa _{2}) \bigr), \qquad \gamma _{E(\kappa _{1}), E(\kappa _{2})}(t)\in B, \end{aligned}$$
  $\forall \kappa _{1},\kappa _{2}\in B$ and $t\in [0,1]$.
2. a geodesic local E-invex (GLEI) with respect to *η* if there is $u(\kappa _{1},\kappa _{2})\in (0,1 ] $ such that $\forall t\in [0,u (\kappa _{1},\kappa _{2})] $,
  1 $$ \gamma _{E(\kappa _{1}),E(\kappa _{2})}(t)\in B \quad \forall \kappa _{1},\kappa _{2}\in B. $$
3. a geodesic local starshaped E-convex if there is a map *E* such that, corresponding to each pair of points $\kappa _{1},\kappa _{2}\in A $, there is a maximal positive number $u(\kappa _{1},\kappa _{2})\leq 1 $ such as
  2 $$ \gamma _{E(\kappa _{1}),E(\kappa _{2})}\in A, \quad \forall t\in \bigl[0, u(\kappa _{1},\kappa _{2})\bigr]. $$

### Definition 2.2

A function $f: A\subset \aleph \longrightarrow \mathbb{R} $ is said to be

1. a geodesic E-preinvex (GEP) on $A\subset \aleph $ with respect to *η* if *A* is a GEI set and
  $$ f \bigl(\gamma _{E(\kappa _{1}),E(\kappa _{2})}(t) \bigr)\leq t f\bigl(E( \kappa _{1}) \bigr)+(1-t)f\bigl(E(\kappa _{2})\bigr) , \quad \forall \kappa _{1},\kappa _{2}\in A, t\in [0,1]; $$
2. a geodesic semi E-preinvex (GSEP) on *A* with respect to *η* if *A* is a GEI set and
  $$ f \bigl(\gamma _{E(\kappa _{1}),E(\kappa _{2})}(t) \bigr)\leq t f(\kappa _{1})+(1-t)f( \kappa _{2}) , \quad \forall \kappa _{1},\kappa _{2}\in A, t\in [0,1]. $$
3. a geodesic local E-preinvex (GLEP) on $A\subset \aleph $ with respect to *η* if, for any $\kappa _{1},\kappa _{2}\in A $, there exists $0< v(\kappa _{1},\kappa _{2})\leq u(\kappa _{1},\kappa _{2}) $ such that *A* is a GLEI set and
  $$ f \bigl(\gamma _{E(\kappa _{1}),E(\kappa _{2})}(t) \bigr)\leq t f\bigl(E( \kappa _{1}) \bigr)+(1-t)f\bigl(E(\kappa _{2})\bigr) , \quad \forall t\in \bigl[0,v( \kappa _{1},\kappa _{2})\bigr]. $$

### Definition 2.3

A function $f:\aleph \longrightarrow \mathbb{R} $ is a geodesic semilocal E-convex (GSLEC) on a geodesic local starshaped E-convex set $B\subset \aleph $ if, for each pair of $\kappa _{1},\kappa _{2}\in B $ (with a maximal positive number $u(\kappa _{1},\kappa _{2})\leq 1 $ satisfying 2), there exists a positive number $v(\kappa _{1},\kappa _{2})\leq u(\kappa _{1},\kappa _{2}) $ satisfying

$$ f \bigl(\gamma _{E(\kappa _{1}), E(\kappa _{2})}(t) \bigr)\leq t f( \kappa _{1})+(1-t)f( \kappa _{2}) , \quad \forall t\in \bigl[0,v(\kappa _{1}, \kappa _{2})\bigr]. $$

### Remark 2.1

Every GEI set with respect to *η* is a GLEI set with respect to *η*, where $u(\kappa _{1},\kappa _{2})=1$, $\forall \kappa _{1},\kappa _{2}\in \aleph $. On the other hand, their converses are not necessarily true, and we can see that in the next example.

### Example 2.1

Put $A= [ \left . -4,-1 ) \right . \cup [1,4] $,

$$\begin{aligned} &E(\kappa ) = \textstyle\begin{cases} \kappa ^{2} & \mbox{if } \vert \kappa \vert \leq 2, \\ -1 & \mbox{if } \vert \kappa \vert > 2; \end{cases}\displaystyle \\ &\eta (\kappa ,\iota ) = \textstyle\begin{cases} \kappa -\iota & \mbox{if } \kappa \geqslant 0, \iota \geqslant 0 \mbox{ or } \kappa \leq 0, \iota \leq 0 , \\ -1-\iota & \mbox{if } \kappa >0, \iota \leq 0 \mbox{ or } \kappa \geqslant 0 ,\iota < 0, \\ 1-\iota & \mbox{if } \kappa < 0, \iota \geqslant 0 \mbox{ or } \kappa \leq 0, \iota >0; \end{cases}\displaystyle \\ &\gamma _{\kappa ,\iota }(t) = \textstyle\begin{cases} \iota +t(\kappa -l) & \mbox{if } \kappa \geqslant 0, \iota \geqslant 0 \mbox{ or } \kappa \leq 0, \iota \leq 0 , \\ \iota +t(-1-\iota ) & \mbox{if } \kappa >0, \iota \leq 0 \mbox{ or } \kappa \geqslant 0 ,\iota < 0, \\ \iota +t(1-\iota ) & \mbox{if } \kappa < 0, \iota \geqslant 0 \mbox{ or } \kappa \leq 0, \iota >0. \end{cases}\displaystyle \end{aligned}$$

Hence *A* is a GLEI set with respect to *η*. However, when $\kappa =3$, $\iota =0 $, there is $t_{1}\in [0,1] $ such that $\gamma _{E(\kappa ),E(\iota )}(t_{1})=-t_{1} $, then if $t_{1}=1 $, we obtain $\gamma _{E(\kappa ),E(\iota )}(t_{1})\notin A $.

### Definition 2.4

A function $f: \aleph \longrightarrow \mathbb{R} $ is GSLEP on $B\subset \aleph $ with respect to *η* if, for any $\kappa _{1},\kappa _{2}\in B $, there is $0< v(\kappa _{1},\kappa _{2})\leq u(\kappa _{1},\kappa _{2})\leq 1 $ such that *B* is a GLEI set and

3 $$ f \bigl(\gamma _{E(\kappa _{1}),E(\kappa _{2})}(t) \bigr)\leq t f(\kappa _{1})+(1-t)f(\kappa _{2}) , \quad \forall t\in \bigl[0,v( \kappa _{1},\kappa _{2})\bigr]. $$

If

$$ f \bigl(\gamma _{E(\kappa _{1}),E(\kappa _{2})}(t) \bigr)\geqslant t f( \kappa _{1})+(1-t)f(\kappa _{2}) , \quad \forall t\in \bigl[0,v( \kappa _{1},\kappa _{2})\bigr], $$

then *f* is GSLEP on *B*.

### Remark 2.2

Any GSLEC function is a GSLEP function. Also, any GSEP function with respect to *η* is a GSLEP function. On the other hand, their converses are not necessarily true. The next example shows SLGEP, which is neither a GSLEC function nor a GSEP function.

### Example 2.2

Assume that $E: \mathbb{R}\longrightarrow \mathbb{R} $ is given as

$$\begin{aligned} E(m) =& \textstyle\begin{cases} 0 & \mbox{if } m< 0, \\ 1 & \mbox{if } 1< m\leq 2, \\ m & \mbox{if } 0\leq m\leq 1 \mbox{ or } m>2; \end{cases}\displaystyle \end{aligned}$$

and the map $\eta : \mathbb{R}\times \mathbb{R}\longrightarrow \mathbb{R} $ is defined as

$$\begin{aligned} \eta (m,n) =& \textstyle\begin{cases} 0 & \mbox{if } m= n, \\ 1-m & \mbox{if } m\neq n ; \end{cases}\displaystyle \end{aligned}$$

also,

$$\begin{aligned} \gamma _{m,n}(t) =& \textstyle\begin{cases} n &\mbox{if } m= n, \\ n+t(1-m) &\mbox{if } m\neq n. \end{cases}\displaystyle \end{aligned}$$

Assume that $h: \mathbb{R}\longrightarrow \mathbb{R} $, where

$$\begin{aligned} h(m) =& \textstyle\begin{cases} 0 & \mbox{if } 1< m\leq 2, \\ 1 & \mbox{if } m>2, \\ -m+1 & \mbox{if } 0\leq m\leq 1, \\ -m+2 & \mbox{if } m< 0; \end{cases}\displaystyle \end{aligned}$$

and since $\mathbb{R} $ is a geodesic local starshaped E-convex set and a geodesic local E-invex set with respect to *η*. Then *h* is a GSLEP on $\mathbb{R} $ with respect to *η*. However, when $m_{0}=2$, $n_{0}=3 $ and for any $v\in (0,1 ] $, there is a sufficiently small $t_{0}\in (0,v ] $ such that

$$ h \bigl(\gamma _{E(m_{0}),E(n_{0})}(t_{0}) \bigr)=1>(1-t_{0})=t_{0}h(m _{0})+(1-t_{0})h(n_{0}) . $$

Then $h(m) $ is not a GSLEC function on $\mathbb{R} $.

Similarly, taking $m_{1}=1$, $n_{1}=4 $, we have

$$ h \bigl(\gamma _{E(m_{1}),E(n_{1})}(t_{1}) \bigr)=1>(1-t_{1})=t_{1}h(m _{1})+(1-t_{1})h(n_{1}) $$

for some $t_{1}\in [0,1] $.

Hence, $h(m) $ is not a GSEP function on $\mathbb{R} $ with respect to *η*.

### Definition 2.5

A function $h:S\subset \aleph \longrightarrow \mathbb{R} $, where *S* is a GLEI set, is said to be a geodesic quasi-semilocal E-preinvex (GqSLEP) (with respect to *η*) if, for all $\kappa _{1},\kappa _{2}\in S $ satisfying $h(\kappa _{1})\leq h( \kappa _{2}) $, there is a positive number $v(\kappa _{1},\kappa _{2}) \leq u(\kappa _{1},\kappa _{2}) $ such that

$$ h \bigl(\gamma _{E(\kappa _{1}),E(\kappa _{2})}(t) \bigr) \leq h(\kappa _{2}),\quad \forall t\in \bigl[0,v(\kappa _{1},\kappa _{2})\bigr]. $$

### Definition 2.6

A function $h:S\subset \aleph \longrightarrow \mathbb{R} $, where *S* is a GLEI set, is said to be a geodesic pseudo-semilocal E-preinvex (GpSLEP) (with respect to *η*) if, for all $\kappa _{1},\kappa _{2}\in S $ satisfying $h(\kappa _{1})< h(\kappa _{2}) $, there are positive numbers $v(\kappa _{1},\kappa _{2})\leq u( \kappa _{1},\kappa _{2}) $ and $w(\kappa _{1},\kappa _{2}) $ such that

$$ h \bigl(\gamma _{E(\kappa _{1}),E(\kappa _{2})}(t) \bigr) \leq h(\kappa _{2})-t w( \kappa _{1},\kappa _{2}),\quad \forall t\in \bigl[0,v(\kappa _{1},\kappa _{2})\bigr]. $$

### Remark 2.3

Every GSLEP on a GLEI set with respect to *η* is both a GqELEP function and a GpSLEP function.

### Definition 2.7

A function $h:S\longrightarrow \mathbb{R} $ is called a geodesic E-*η*- semidifferentiable at $\kappa ^{*} \in S $, where $S\subset \aleph $ is a GLEI set with respect to *η*, if $E(\kappa ^{*})=\kappa ^{*} $ and

$$ h'_{+} \bigl(\gamma _{\kappa ^{*},E(\kappa )}(t) \bigr)= \lim _{t\longrightarrow 0^{+}} \frac{1}{t} \bigl[h \bigl(\gamma _{\kappa ^{*},E( \kappa )}(t) \bigr) -h\bigl(\kappa ^{*}\bigr) \bigr] $$

exist for every $\kappa \in S$.

### Remark 2.4

1. If $\aleph =\mathbb{R}^{n} $, then the geodesic E-*η*- semidifferentiable is E-*η*-semidifferentiable [11].
2. If $\aleph =\mathbb{R}^{n} $ and $E=I $, then the geodesic E-*η*-semidifferentiable is the *η*-semidifferentiability [23].
3. If $\aleph =\mathbb{R}^{n} $, $E=I $, and $\eta (\kappa ,\kappa ^{*})=\kappa -\kappa ^{*} $, then the geodesic E-*η*-semidifferentiable is the semidifferentiability [11].

### Lemma 2.1

1. *Assume that* *h* *is a GSLEP* (*E*-*preconcave*) *and a geodesic E*-*η*-*semidifferentiable at* $\kappa ^{*}\in S\subset \aleph $, *where* *S* *is a GLEI set with respect to* *η*. *Then*
  $$ h(\kappa )-h\bigl(\kappa ^{*}\bigr)\geqslant (\leq ) h'_{+}\bigl( \gamma _{\kappa ^{*},E(\kappa )}(t)\bigr),\quad \forall \kappa \in S. $$
2. *Let* *h* *be a GqSLEP* (*GpSLEP*) *and a geodesic E*-*η*-*semidifferentiable at* $\kappa ^{*}\in S\subset \aleph $, *where* *S* *is a LGEI set with respect to* *η*. *Hence*
  $$ h(\kappa )\leq (< ) h\bigl(\kappa ^{*}\bigr)\quad \Rightarrow\quad h'_{+}\bigl( \gamma _{\kappa ^{*},E(\kappa )}(t)\bigr)\leq (< )0, \quad \forall \kappa \in S. $$

The above lemma is proved directly by using definitions (Definition 2.4, Definition 2.5, Definition 2.6, and Definition 2.4).

### Theorem 2.1

*Let* $f: S\subset \aleph \longrightarrow \mathbb{R} $ *be a GLEP function on a GLEI set* *S* *with respect to* *η*, *then* *f* *is a GSLEP function iff* $f(E(\kappa ))\leq f( \kappa )$, $\forall \kappa \in S $.

### Proof

Assume that *f* is a GSLEP function on set *S* with respect to *η*, then $\forall \kappa _{1},\kappa _{2}\in S $, there is a positive number $v(\kappa _{1},\kappa _{2})\leq u(\kappa _{1},\kappa _{2}) $ where

$$ f\bigl(\gamma _{E(\kappa _{1}),E(\kappa _{2})}(t)\bigr)\leq tf(\kappa _{2})+(1-t)f( \kappa _{1}),\quad t\in \bigl[0,v(\kappa _{1},\kappa _{2})\bigr]. $$

By letting $t=0 $, then $f(E(\kappa _{1}))\leq f(\kappa _{1})$, $\forall \kappa _{1}\in S $.

Conversely, consider that *f* is a GLEP function on a GLEI set *S*, then for any $\kappa _{1},\kappa _{2}\in S $, there exist $u(\kappa _{1},\kappa _{2}) \in (0,1 ] $ (1) and $v(\kappa _{1},\kappa _{2}) \in (0,u(\kappa _{1},\kappa _{2}) ] $ such that

$$ f\bigl(\gamma _{E(\kappa _{1}),E(\kappa _{2})}(t)\bigr)\leq tf\bigl(E(\kappa _{1}) \bigr)+(1-t)f\bigl(E( \kappa _{2})\bigr),\quad t\in \bigl[0,v(\kappa _{1},\kappa _{2})\bigr]. $$

Since $f(E(\kappa _{1})) \leq f(\kappa _{1})$, $\forall \kappa _{1}\in S$, then

$$ f\bigl(\gamma _{E(\kappa _{1}),E(\kappa _{2})}(t)\bigr)\leq tf(\kappa _{1})+(1-t)f( \kappa _{2}),\quad t\in \bigl[0,v(\kappa _{1},\kappa _{2})\bigr]. $$

 □

### Definition 2.8

The set $\omega = \lbrace (\kappa , \alpha ):\kappa \in B\subset \aleph , \alpha \in \mathbb{R} \rbrace $ is said to be a GLEI set with respect to *η* corresponding to ℵ if there are two maps *η*, *E* and a maximal positive number $u((\kappa _{1},\alpha _{1}), (\kappa _{2}, \alpha _{2}))\leq 1 $ for each $(\kappa _{1},\alpha _{1}), (\kappa _{2}, \alpha _{2})\in \omega $ such that

$$ \bigl(\gamma _{E(\kappa _{1}),E(\kappa _{2})}(t),t\alpha _{1}+(1-t)\alpha _{2} \bigr)\in \omega ,\quad \forall t\in \bigl[0,u\bigl((\kappa _{1},\alpha _{1}), (\kappa _{2},\alpha _{2})\bigr) \bigr]. $$

### Theorem 2.2

*Let* $B\subset \aleph $ *be a GLEI set with respect to* *η*. *Then* *f* *is a GSLEP function on* *B* *with respect to* *η* *iff its epigraph*

$$ \omega _{f}= \bigl\lbrace (\kappa _{1},\alpha ):\kappa _{1}\in B, f( \kappa _{1})\leq \alpha , \alpha \in \mathbb{R} \bigr\rbrace $$

*is a GLEI set with respect to* *η* *corresponding to* ℵ.

### Proof

Suppose that *f* is a GSLEP on *B* with respect to *η* and $(\kappa _{1},\alpha _{1}), (\kappa _{2},\alpha _{2})\in \omega _{f} $, then $\kappa _{1},\kappa _{2}\in B$, $f(\kappa _{1})\leq \alpha _{1}$, $f(\kappa _{2})\leq \alpha _{2} $. By applying Definition 2.1, we obtain $\gamma _{E(\kappa _{1}),E(\kappa _{2})}(t)\in B$, $\forall t\in [0, u(\kappa _{1},\kappa _{2}) ]$.

Moreover, there is a positive number $v(\kappa _{1},\kappa _{2})\leq u( \kappa _{1},\kappa _{2}) $ such that

$$ f \bigl(\gamma _{E(\kappa _{1}),E(\kappa _{2})}(t), t\alpha _{1}+(1-t) \alpha _{2} \bigr)\in \omega _{f},\quad \forall t\in \bigl[0,v(\kappa _{1},\kappa _{2})\bigr]. $$

Conversely, if $\omega _{f} $ is a GLEI set with respect to *η* corresponding to ℵ, then for any points $(\kappa _{1},f(\kappa _{1})) , (\kappa _{2},f(\kappa _{2}))\in \omega _{f}$, there is a maximal positive number $u((\kappa _{1},f(\kappa _{1})), (\kappa _{2},f(\kappa _{2}))\leq 1 $ such that

$$ \bigl( \gamma _{E(\kappa _{1}),E(\kappa _{2})}(t), tf(\kappa _{1}) +(1-t)f( \kappa _{2}) \bigr) \in \omega _{f},\quad \forall t\in \bigl[0, u\bigl( \bigl(\kappa _{1},f(\kappa _{1})\bigr),\bigl(\kappa _{2},f(\kappa _{2})\bigr)\bigr) \bigr]. $$

That is, $\gamma _{E(\kappa _{1}),E(\kappa _{2})}(t) \in B$,

$$ f \bigl(\gamma _{E(\kappa _{1}),E(\kappa _{2})}(t) \bigr)\leq tf(\kappa _{1}) +(1-t)f( \kappa _{2}), \quad t\in \bigl[0,u\bigl(\bigl(\kappa _{1},f( \kappa _{1})\bigr),\bigl(\kappa _{2},f(\kappa _{2}) \bigr)\bigr) \bigr]. $$

Thus, *B* is a GLEI set and *f* is a GSLEP function on *B*. □

### Theorem 2.3

*If* *f* *is a GSLEP function on a GLEI set* $B\subset \aleph $ *with respect to* *η*, *then the level* $K_{\alpha }= \lbrace \kappa _{1}\in B: f(\kappa _{1})\leq \alpha \rbrace $ *is a GLEI set for any* $\alpha \in \mathbb{R} $.

### Proof

For any $\alpha \in \mathbb{R}$ and $\kappa _{1},\kappa _{2} \in K_{\alpha } $, then $\kappa _{1},\kappa _{2}\in B $ and $f(\kappa _{1})\leq \alpha $, $f(\kappa _{2})\leq \alpha $. Since *B* is a GLEI set, then there is a maximal positive number $u(\kappa _{1},\kappa _{2})\leq 1 $ such that

$$ \gamma _{E(\kappa _{1}),E(\kappa _{2})}(t)\in B, \quad \forall t\in \bigl[0,u(\kappa _{1},\kappa _{2}) \bigr] . $$

In addition, since *f* is GSLEP, there is a positive number $v(\kappa _{1},\kappa _{2})\leq u(y_{1},y_{2}) $ such that

$$\begin{aligned} f \bigl(\gamma _{E(\kappa _{1}),E(\kappa _{2})}(t) \bigr) \leq & t f( \kappa _{1}) +(1-t)f(\kappa _{2}) \\ \leq & t\alpha +(1-t)\alpha \\ =& \alpha , \quad \forall t\in \bigl[0,v(\kappa _{1},\kappa _{2}) \bigr]. \end{aligned}$$

That is, $\gamma _{E(\kappa _{1}),E(\kappa _{2})}(t)\in K_{\alpha }$, $\forall t\in [0,v(\kappa _{1},\kappa _{2}) ] $. Therefore, $K_{\alpha } $ is a GLEI set with respect to *η* for any $\alpha \in \mathbb{R} $. □

### Theorem 2.4

*Let* $f:B\subset \aleph \longrightarrow \mathbb{R} $, *where* *B* *is a GLEI*. *Then* *f* *is a GSLEP function with respect to* *η* *if*,*f for each pair of points* $\kappa _{1},\kappa _{2}\in B $, *there is a positive number* $v(\kappa _{1},\kappa _{2}) \leq u(\kappa _{1},\kappa _{2})\leq 1 $ *such that*

$$ f \bigl(\gamma _{E(\kappa _{1}),E(\kappa _{2})}(t) \bigr) \leq t \alpha +(1-t)\beta , \quad \forall t\in \bigl[0,v(\kappa _{1},\kappa _{2}) \bigr]. $$

### Proof

Let $\kappa _{1},\kappa _{2}\in B $ and $\alpha ,\beta \in \mathbb{R} $ such that $f(\kappa _{1})<\alpha $ and $f(\kappa _{2})<\beta $. Since *B* is GLEI, there is a maximal positive number $u(\kappa _{1}, \kappa _{2})\leq 1 $ such that

$$ \gamma _{E(\kappa _{1}),E(\kappa _{2})}(t) \in B , \quad \forall t\in \bigl[0,u(\kappa _{1},\kappa _{2}) \bigr]. $$

In addition, there is a positive number $v(\kappa _{1},\kappa _{2}) \leq u(\kappa _{1},\kappa _{2}) $, where

$$ f \bigl(\gamma _{E(\kappa _{1}),E(\kappa _{2})}(t) \bigr) \leq t \alpha +(1-t)\beta , \quad \forall t\in \bigl[0,v(\kappa _{1},\kappa _{2}) \bigr]. $$

Conversely, let $(\kappa _{1},\alpha ) \in \omega _{f} $ and $(\kappa _{2},\beta ) \in \omega _{f} $, then $\kappa _{1},\kappa _{2} \in B $, $f(\kappa _{1})<\alpha $, and $f(\kappa _{2})<\beta $. Hence, $f(\kappa _{1})<\alpha +\varepsilon $ and $f(\kappa _{2})<\beta + \varepsilon $ hold for any $\varepsilon >0 $. According to the hypothesis for $\kappa _{1},\kappa _{2}\in B $, there is a positive number $v(\kappa _{1},\kappa _{2})\leq u(\kappa _{1},\kappa _{2})\leq 1 $ such that

$$ f \bigl(\gamma _{E(\kappa _{1}),E(\kappa _{2})}(t) \bigr) \leq t \alpha +(1-t)\beta +\varepsilon , \quad \forall t\in \bigl[0,v(\kappa _{1},\kappa _{2}) \bigr]. $$

Let $\varepsilon \longrightarrow 0^{+} $, then

$$ f \bigl(\gamma _{E(\kappa _{1}),E(\kappa _{2})}(t) \bigr) \leq t \alpha +(1-t)\beta , \quad \forall t\in \bigl[0,v(\kappa _{1},\kappa _{2}) \bigr]. $$

That is, $(\gamma _{E(\kappa _{1}),E(\kappa _{2})}(t) , t \alpha +(1-t) \beta ) \in \omega _{f} $, $\forall t\in [0,v(\kappa _{1},\kappa _{2}) ]$.

Therefore, $\omega _{f} $ is a GLEI set corresponding to ℵ. From Theorem 2.2 it follows that *f* is a GSLEP on *B* with respect to *η*. □

## Optimality criteria

In this section, let us consider the nonlinear fractional multiobjective programming problem

$$\begin{aligned} (\mbox{VFP}) \textstyle\begin{cases} \mbox{minimize } \frac{f(\kappa )}{g(\kappa )}= (\frac{f_{1}(\kappa )}{g_{1}( \kappa )},\ldots ,\frac{f_{p}(\kappa )}{g_{p}(\kappa )} ), \\ \mbox{subject to } h_{j}(\kappa )\leq 0,\quad j\in Q={1,2,\ldots, q} \\ \kappa \in K_{0}; \end{cases}\displaystyle \end{aligned}$$

where $K_{0}\subset \aleph $ is a GLEI set and $g_{i}(\kappa )>0$, $\forall \kappa \in K_{0} $, $i\in P={1,2,\ldots , p} $.

Let $f=(f_{1},f_{2},\ldots , f_{p})$, $g=(g_{1},g_{2},\ldots ,g_{p}) $, and $h=(h_{1},h_{2},\ldots ,h_{q}) $

and denote that $K= \lbrace \kappa :h_{j}(\kappa )\leq 0, j \in Q, \kappa \in K_{0} \rbrace $, the feasible set of problem (VFP).

For $\kappa ^{*}\in K $, we put

$$ Q\bigl(\kappa ^{*}\bigr)= \bigl\lbrace j:h_{j}\bigl(\kappa ^{*}\bigr)= 0, j\in Q \bigr\rbrace , \qquad L\bigl(\kappa ^{*}\bigr)=\frac{Q}{Q(\kappa ^{*})}. $$

We also formulate the nonlinear multiobjective programming problem as follows:

$$\begin{aligned} (\mbox{VFP}_{\lambda }) \textstyle\begin{cases} \mbox{minimize } ( f_{1}(\kappa )-\lambda _{1}g_{1}(\kappa ),\ldots f_{p}(\kappa )-\lambda _{p}g_{p}(\kappa ) ), \\ \mbox{subject to } h_{j}(\kappa )\leq 0,\quad j\in Q={1,2,\ldots, q} \\ \kappa \in K_{0}; \end{cases}\displaystyle \end{aligned}$$

where $\lambda =(\lambda _{1},\lambda _{2},\ldots ,\lambda _{p})\in \mathbb{R}^{p} $.

The following lemma connects the weak efficient solutions for (VFP) and ($\mathrm{VFP}_{\lambda } $).

### Lemma 3.1

*A point* $\kappa ^{*} $ *is a weak efficient solution for* ($\mathrm{VFP}_{\lambda } $) *iff* $\kappa ^{*} $ *is a weak efficient solution for* ($\mathrm{VFP}^{*}_{\lambda } $), *where* $\lambda ^{*}=(\lambda ^{*}_{1}, \ldots ,\lambda ^{*}_{p} )= (\frac{f_{1}(\kappa ^{*})}{g_{1}(\kappa ^{*})},\ldots ,\frac{f_{p}(\kappa ^{*})}{g_{p}(\kappa ^{*})} ) $.

### Proof

Assume that there is a feasible point $\kappa \in K $, where

$$ f_{i}(\kappa )-\lambda ^{*}_{i}g_{i}( \kappa )< f_{i}\bigl(\kappa ^{*}\bigr)-\lambda ^{*}_{i}g_{i}\bigl(\kappa ^{*}\bigr),\quad \forall i\in Q $$

⟹

$$ f_{i}(\kappa )< \frac{f_{i}(\kappa ^{*})}{g_{i}(\kappa ^{*})g_{i}(\kappa )} $$

⟹

$$ \frac{f_{i}(\kappa )}{g_{i}(\kappa )}< \frac{f_{i}(\kappa ^{*})}{g_{i}( \kappa ^{*})}, $$

which is a contradiction to the weak efficiency of $\kappa ^{*} $ for (VFP).

Presently, let us take $\kappa \in K $ as a feasible point such that

$$ \frac{f_{i}(\kappa )}{g_{i}(\kappa )}< \frac{f_{i}(\kappa ^{*})}{g_{i}( \kappa ^{*})}= \lambda ^{*}_{i}, $$

then $f_{i}(\kappa )-\lambda ^{*}_{i}g_{i}(\kappa )<0=f_{i}(\kappa ^{*})-\lambda ^{*}_{i}g_{i}(\kappa ^{*})$, $\forall i\in Q $, which is again a contradiction to the weak efficiency of $\kappa ^{*} $ for ($\mathrm{VFP} ^{*}_{\lambda } $). □

Next, some sufficient optimality conditions for the problem (VFP) are established.

### Theorem 3.1

*Let* $\bar{\kappa }\in K$, $E(\bar{\kappa })=\bar{ \kappa } $ *and* *f*, *h* *be GSLEP and* *g* *be a geodesic semilocal E*-*preincave*, *and they are all geodesic E*-*η*- *semidifferentiable at* *κ̄*. *Further*, *assume that there are* $\zeta ^{o}= (\zeta ^{o}_{i}, i=1,\ldots ,p )\in \mathbb{R}^{p} $ *and* $\xi ^{o}= (\xi ^{o}_{j}, j=1,\ldots ,m )\in \mathbb{R} ^{m} $ *such that*

4 $$\begin{aligned} &\zeta ^{o}_{i}f'_{i+} \bigl(\gamma _{\bar{\kappa },E(\widehat{\kappa })}(t) \bigr)+\xi ^{o}_{j} h'_{j+} \bigl(\gamma _{\bar{\kappa },E( \widehat{\kappa })}(t) \bigr)\geqslant 0\quad \forall \kappa \in K, t \in [0,1], \end{aligned}$$

5 $$\begin{aligned} &g'_{i+} \bigl(\gamma _{\bar{\kappa },E(\kappa )}(t) \bigr)\leq 0,\quad \forall \kappa \in K, i\in P, \end{aligned}$$

6 $$\begin{aligned} &\xi ^{o}h(\bar{\kappa })=0 \end{aligned}$$

7 $$\begin{aligned} &\zeta ^{o}\geqslant 0 ,\qquad \xi ^{o}\geqslant 0. \end{aligned}$$

*Then* *κ̄* *is a weak efficient solution for* (VFP).

### Proof

By contradiction, let *κ̄* be not a weak efficient solution for (VFP), then there exists a point $\widehat{\kappa }\in K $ such that

8 $$ \frac{f_{i}(\widehat{\kappa })}{g_{i}(\widehat{\kappa })}< \frac{f_{i}(\bar{ \kappa })}{g_{i}(\bar{\kappa })},\quad i\in P. $$

By the above hypotheses and Lemma 3.1, we have

9 $$\begin{aligned} &f_{i}(\widehat{\kappa })-f_{i}(\bar{\kappa })\geqslant f'_{i+} \bigl(\gamma _{\bar{\kappa },E(\widehat{\kappa })}(t) \bigr) ,\quad i\in P \end{aligned}$$

10 $$\begin{aligned} &g_{i}(\widehat{\kappa })-g_{i}(\bar{\kappa })\leq g'_{i+} \bigl(\gamma _{\bar{ \kappa },E(\widehat{\kappa })}(t) \bigr) ,\quad i \in P \end{aligned}$$

11 $$\begin{aligned} &h_{i}(\widehat{\kappa })-h_{i}(\bar{\kappa })\geqslant h'_{j+} \bigl(\gamma _{\bar{\kappa },E(\widehat{\kappa })}(t) \bigr) ,\quad j\in Q. \end{aligned}$$

Multiplying (9) by $\zeta ^{o}_{i} $ and (11) by $\xi ^{o}_{j} $, we get

12 $$\begin{aligned} & \sum_{i=1}^{p} \zeta ^{o}_{i} \bigl(f_{i}(\widehat{\kappa })-f_{i}(\bar{ \kappa }) \bigr) + \sum_{j=1}^{m} \xi ^{o}_{j} \bigl(h_{j}( \widehat{\kappa })-h_{j}(\bar{\kappa }) \bigr) \\ &\quad \geqslant \zeta ^{o}_{i} f'_{i+} \bigl(\gamma _{\bar{\kappa },E( \widehat{\kappa })}(t) \bigr) +\xi ^{o}_{j} h'_{j+} \bigl(\gamma _{\bar{ \kappa },E(\widehat{\kappa })}(t) \bigr) \geqslant 0. \end{aligned}$$

Since $\widehat{\kappa }\in K, \xi ^{o}\geqslant 0 $ by (6) and (12), we have

13 $$ \sum_{i=1}^{p} \zeta ^{o}_{i} \bigl(f_{i}(\widehat{\kappa })-f_{i}(\bar{ \kappa }) \bigr)\geqslant 0. $$

Utilizing (7) and (13), there is at least $i_{0} $ ($1\leq i_{0}\leq p $) such that

14 $$ f_{i_{0}}(\widehat{\kappa })\geqslant f_{i_{0}}( \bar{\kappa }). $$

On the other hand, (5) and (10) imply

15 $$ g_{i}(\widehat{\kappa })\leq g_{i}(\bar{ \kappa }),\quad i\in P. $$

By using (14), (15), and $g>0 $, we have

16 $$ \frac{f_{i_{0}}(\widehat{\kappa })}{g_{i_{0}}(\widehat{\kappa })} \geqslant \frac{f_{i_{0}}(\bar{\kappa })}{g_{i_{0}}(\bar{\kappa })}, $$

which is a contradiction to 8, then the proof of the theorem is completed. □

Similarly we can prove the next theorem.

### Theorem 3.2

*Consider that* $\bar{\kappa }\in B$, $E(\bar{ \kappa })=\bar{\kappa } $ *and* *f*, *h* *are geodesic E*-*η*-*semidifferentiable at κ̄*. *If there exist* $\zeta ^{o} \in \mathbb{R}^{n} $ *and* $\xi ^{o}\in \mathbb{R}^{m} $ *such that conditions* (4)*–*(7) *hold and* $\zeta ^{o}f(x)+\xi ^{o}h(x) $ *is a GSLEP function*, *then* *κ̄* *is a weak efficient solution for* (VFP).

### Theorem 3.3

*Consider that* $\bar{\kappa }\in B$, $E(\bar{ \kappa })=\bar{\kappa } $ *and* $\lambda _{i}^{o}=\frac{f_{i}(\bar{ \kappa })}{g_{i}(\bar{\kappa })}$ ($i\in P$) *are all pSLGEP functions and* $h_{j}(\kappa )$ ($j\in \aleph (\bar{\kappa })$) *are all GqSLEP functions and* *f*, *g*, *h* *are all geodesic E*-*η*-*semidifferentiable at* *κ̄*. *If there are* $\zeta ^{o}\in \mathbb{R}^{p} $ *and* $\xi ^{o}\in \mathbb{R}^{m} $ *such that*

17 $$\begin{aligned} &\sum_{i=1}^{p}\zeta _{i}^{o} \bigl(f'_{i+} \bigl( \gamma _{\bar{\kappa },E(\kappa )}(t) \bigr) -\lambda _{i}^{o}g'_{i+} \bigl( \gamma _{\bar{\kappa },E(\kappa )}(t) \bigr) \bigr) +\xi ^{o}h'_{i+} \bigl( \gamma _{\bar{\kappa },E(\kappa )}(t) \bigr) \geqslant 0 \end{aligned}$$

18 $$\begin{aligned} &\xi ^{o}h(\bar{\kappa })=0, \end{aligned}$$

19 $$\begin{aligned} &\zeta ^{o}\geqslant 0,\qquad \xi ^{o}\geqslant 0, \end{aligned}$$

*then* *κ̄* *is a weak efficient solution for* (VFP).

### Proof

Assume that *κ̄* is not a weak efficient solution for (VFP). Therefore, there exists $\kappa ^{*}\in B $, which yields

$$ \frac{f_{i}(\kappa ^{*})}{g_{i}(\kappa ^{*})}< \frac{f_{i}(\bar{\kappa })}{g _{i}(\bar{\kappa })}. $$

Then

$$ f_{i}\bigl(\kappa ^{*}\bigr)-\lambda _{i}^{o}g_{i} \bigl(\kappa ^{*}\bigr)< 0, \quad i\in P, $$

which means that

$$ f_{i}\bigl(\kappa ^{*}\bigr)-\lambda _{i}^{o}g_{i} \bigl(\kappa ^{*}\bigr)< f_{i}(\bar{\kappa })-\lambda _{i}^{o}g_{i}(\bar{\kappa })< 0, \quad i\in P. $$

By the pSLGEP of $( f_{i}(\kappa )-\lambda _{i}^{o}g_{i}(\kappa ) )$ ($i\in P$) and Lemma 2.1, we have

$$ f'_{i+} \bigl( \gamma _{\bar{\kappa },E(\kappa )}(t) \bigr) -\lambda _{i}^{o}g'_{i+} \bigl( \gamma _{\bar{\kappa },E(\kappa )}(t) \bigr) , \quad i\in P. $$

Utilizing $\zeta ^{o}\geqslant 0 $, then

20 $$ \sum_{i=1}^{p}\zeta _{i}^{o} \bigl(f'_{i+} \bigl( \gamma _{\bar{\kappa },E(\kappa )}(t) \bigr) -\lambda _{i}^{o}g'_{i+} \bigl( \gamma _{\bar{\kappa },E(\kappa )}(t) \bigr) \bigr)< 0. $$

For $h(\kappa ^{*})\leq 0 $ and $h_{j}(\bar{\kappa })= 0$, $j \in \aleph (\bar{\kappa }) $, we have $h_{j}(\kappa ^{*})\leq h_{j}(\bar{ \kappa })$, $\forall j\in \aleph (\bar{\kappa })$.

By the GqSLEP of $h_{j} $ and Lemma 2.1, we have

$$ h_{j+} \bigl( \gamma _{\bar{\kappa },E(\kappa )}(t) \bigr) \leq 0, \quad \forall j \in \aleph (\bar{\kappa }). $$

Considering $\xi ^{o}\geqslant 0 $ and $\xi _{j}^{o}= 0$, $j\in \aleph (\bar{\kappa })$, then

21 $$ \sum_{j=1}^{m}\xi _{j}^{o}h'_{j+} \bigl( \gamma _{\bar{\kappa },E(\kappa ^{*})}(t) \bigr) \leq 0. $$

Hence, by (20) and (21), we have

22 $$\begin{aligned} \sum_{i=1}^{p}\zeta _{i}^{o} \bigl(f'_{i+} \bigl( \gamma _{\bar{\kappa },E(\kappa ^{*})}(t) \bigr) - \lambda _{i}^{o}g'_{i+} \bigl( \gamma _{\bar{\kappa },E(\kappa ^{*})}(t) \bigr) \bigr) +\xi ^{o}h'_{i+} \bigl( \gamma _{\bar{\kappa },E(\kappa ^{*})}(t) \bigr) < 0, \end{aligned}$$

which is a contradiction to relation (17) at $\kappa ^{*} \in B $. Therefore, *κ̄* is a weak efficient solution for (VFP). □

### Theorem 3.4

*Consider* $\bar{\kappa }\in B$, $E(\bar{\kappa })=\bar{ \kappa } $ *and* $\lambda _{i}^{o}=\frac{f_{i}(\bar{\kappa })}{g_{i}(\bar{ \kappa })}(i\in P) $. *Also*, *assume that* *f*, *g*, *h* *are geodesic E*-*η*-*semidifferentiable at* *κ̄*. *If there are* $\zeta ^{o}\in \mathbb{R}^{p} $ *and* $\xi ^{o}\in \mathbb{R}^{m} $ *such that conditions* (17)*–*(19) *hold and* $\sum_{i=1}^{p} \zeta ^{o}_{i} (f_{i}(\kappa )-\lambda ^{o}_{i}g_{i}(\kappa ) )+ \xi ^{o}_{\aleph (\bar{\kappa })}h_{\aleph (\bar{\kappa })}(\kappa ) $ *is a GpSLEP function*, *then* *κ̄* *is a weak efficient solution for* (VFP).

### Corollary 3.1

*Let* $\bar{\kappa }\in B$, $E(\bar{\kappa })=\bar{ \kappa } $ *and* $\lambda _{i}^{o}=\frac{f_{i}(\bar{\kappa })}{g_{i}(\bar{ \kappa })}(i\in P) $. *Further*, *let* *f*, $h_{\aleph (\bar{\kappa })}$ *be all GSLEP functions*, *g* *be a geodesic semilocal E*-*preincave function*, *and* *f*, *g*, *h* *be all geodesic E*-*η*- *semidifferentiable at* *κ̄*. *If there exist* $\zeta ^{o}\in \mathbb{R}^{p} $ *and* $\xi ^{o}\in \mathbb{R}^{m} $ *such that conditions* (17)*–*(19) *hold*, *then* *κ̄* *is a weak efficient solution for* (VFP).

The dual problem for (VFP) is formulated as follows:

$$\begin{aligned} (\mathrm{VFD}) \textstyle\begin{cases} \mbox{minimize } (\zeta _{i}, i=1,2,\ldots , p ) , \\ \mbox{subject to } \sum_{i=1}^{p}\alpha _{i} (f'_{i+} ( \gamma _{\lambda ,E( \kappa )}(t) ) -\zeta _{i}g'_{i+} ( \gamma _{\lambda ,E( \kappa )}(t) ) ) +\sum_{j=1}^{m}\beta _{j}h'_{j+} ( \gamma _{\lambda ,E(\kappa )}(t) ) \geqslant 0 \\ \quad \kappa \in K_{0}, t\in [0,1], \\ \quad f_{i}(\lambda )-\zeta _{i}g_{i}(\lambda )\geqslant 0, \quad i\in P,\qquad \beta _{j}h_{j}(\lambda )\geqslant 0, \quad j\in \aleph ; \end{cases}\displaystyle \end{aligned}$$

where $\zeta =(\zeta _{i}, i=1,2,\ldots , p)\geqslant 0$, $\alpha =( \alpha _{i}, i=1,2,\ldots , p)> 0$, $\beta =(\beta _{i}, i=1,2,\ldots , m)\geqslant 0$, $\lambda \in K_{0}$.

Denote the feasible set problem ($VFD $) by $K^{\prime} $.

### Theorem 3.5

(General weak duality)

*Let* $\kappa \in K $, $(\alpha ,\beta ,\lambda ,\zeta )\in K^{\prime} $, *and* $E(\lambda )= \lambda $. *If* $\sum_{i=1}^{p}\alpha _{i}(f_{i}-\zeta _{i}g_{i}) $ *is a GpSLEP function and* $\sum_{j=1}^{m}\beta _{j}h_{j} $ *is a GqSLEP function and they are all geodesic E*-*η*-*semidifferentiable at* *λ*, *then* $\frac{f(\kappa )}{g(\kappa )}\nleq \zeta $.

### Proof

From $\alpha >0 $ and $(\alpha , \beta ,\lambda ,\zeta )\in K^{\prime} $, we have

$$ \sum_{i=1}^{p}\alpha _{i} \bigl(f_{i}(\kappa )-\zeta _{i}g_{i}(\kappa ) \bigr)< 0 \leq \sum_{i=1}^{p}\alpha _{i}\bigl(f_{i}(\lambda )-\zeta _{i}g_{i}( \lambda )\bigr). $$

By the GpSLEP of $\sum_{i=1}^{p}\alpha _{i}(f_{i}-\zeta _{i}g_{i}) $ and Lemma 2.1, we obtain

$$ \Biggl( \sum_{i=1}^{p}\alpha _{i}(f_{i}-\zeta _{i}g_{i}) \Biggr)'_{+} \bigl(\gamma _{\lambda ,E(\kappa )}(t) \bigr) < 0, $$

that is,

$$ \sum_{i=1}^{p}\alpha _{i} (f'_{i+} \bigl( \gamma _{\lambda ,E( \kappa )}(t) \bigr) -\zeta _{i}g'_{i+} \bigl( \gamma _{\lambda ,E( \kappa )}(t) ] \bigr)< 0. $$

Also, from $\beta \geqslant 0$ and $\kappa \in K $, then

$$ \sum_{j=1}^{m}\beta _{j}h_{j}( \kappa )\leq 0 \leq \sum_{j=1}^{m}\beta _{j}h_{j}(\lambda ). $$

Using the GqSLEP of $\sum_{j=1}^{m}\beta _{j}h_{j} $ and Lemma 2.1, one has

$$ \Biggl( \sum_{j=1}^{m}\beta _{j}h_{j} \Biggr)'_{+} \bigl(\gamma _{\lambda ,E( \kappa )}(t) \bigr) \leq 0. $$

Then

$$ \sum_{j=1}^{m}\beta _{j}h'_{j+} \bigl(\gamma _{\lambda ,E(\kappa )}(t) \bigr) \leq 0. $$

Therefore,

$$ \sum_{i=1}^{p}\alpha _{i} \bigl(f'_{i+} \bigl( \gamma _{\lambda ,E( \kappa )}(t) \bigr) - \zeta _{i}g'_{i+} \bigl( \gamma _{\lambda ,E( \kappa )}(t) \bigr) \bigr) +\sum_{j=1}^{m} \beta _{j}h'_{j+} \bigl( \gamma _{\lambda ,E(\kappa )}(t) \bigr) < 0. $$

This is a contradiction to $(\alpha ,\beta ,\lambda ,\zeta )\in K ^{\prime} $. □

### Theorem 3.6

*Consider that* $\kappa \in K $, $(\alpha , \beta ,\lambda ,\zeta )\in K^{\prime} $ *and* $E(\lambda )=\lambda $. *If* $\sum_{i=1}^{p}\alpha _{i}(f_{i}-\zeta _{i}g_{i})+\sum_{j=1}^{m}\beta _{j}h_{j} $ *is a GpSLEP function and a geodesic E*-*η*-*semidifferentiable at* *λ*, *then* $\frac{f(\kappa )}{g( \kappa )}\nleq \zeta $.

### Theorem 3.7

(General converse duality)

*Let* $\bar{\kappa } \in K $ *and* $(\kappa ^{*},\alpha ^{*}, \beta ^{*},\zeta ^{*})\in K^{\prime} $, $E(\kappa ^{*})=\kappa ^{*} $, *where* $\zeta ^{*}= \frac{f(\kappa ^{*})}{g( \kappa ^{*})}=\frac{f(\bar{\kappa })}{g(\bar{\kappa })}=(\zeta ^{*}_{i}, i=1,2,\ldots , p) $. *If* $f_{i}-\zeta _{i}^{*}g_{i} (i\in P)$, $h _{j}(j\in \aleph )$ *are all GSLEP functions and all geodesic E*-*η*-*semidifferentiable at* $\kappa ^{*} $, *then* *κ̄* *is a weak efficient solution for* (VFP).

### Proof

By using the hypotheses and Lemma 2.1, for any $\kappa \in K $, we obtain

$$\begin{aligned} &\bigl( f_{i}(\kappa )-\zeta _{i}^{*}g_{i}( \kappa ) \bigr) - \bigl(f _{i}\bigl(\kappa ^{*}\bigr)-\zeta _{i}^{*}g_{i}\bigl(\kappa ^{*}\bigr) \bigr)\geqslant f'_{i+} \bigl( \gamma _{\kappa ^{*},E(\kappa )}(t) \bigr) -\zeta _{i}g'_{i+} \bigl( \gamma _{\kappa ^{*},E(\kappa )}(t) \bigr) \\ &h_{j}(y)-h_{j}\bigl(\kappa ^{*}\bigr)\geqslant h'_{j+} \bigl( \gamma _{\kappa ^{*},E(\kappa )}(t) \bigr). \end{aligned}$$

Utilizing the first constraint condition for (VFD), $\alpha ^{*}>0, \beta ^{*}\geqslant 0$, $\zeta ^{*}\geqslant 0 $, and the two inequalities above, we have

23 $$\begin{aligned} & \sum_{i=1}^{p}\alpha ^{*}_{i} \bigl( \bigl( f_{i}(\kappa )-\zeta _{i}^{*}g_{i}(\kappa ) \bigr) - \bigl(f_{i}\bigl(\kappa ^{*}\bigr)-\zeta _{i} ^{*}g_{i}\bigl(\kappa ^{*}\bigr) \bigr) \bigr) + \sum_{j=1}^{m}\beta ^{*}_{j} \bigl(h_{j}(\kappa )-h_{j}\bigl(\kappa ^{*}\bigr) \bigr) \\ &\quad \geqslant \sum_{i=1}^{p} \bigl(f'_{i+} \bigl( \gamma _{\kappa ^{*},E(\kappa )}(t) \bigr) - \zeta _{i}g'_{i+} \bigl( \gamma _{\kappa ^{*},E(\kappa )}(t) \bigr) \bigr) \\ &\qquad {}+\sum_{j=1}^{m}\beta ^{*}_{j} h'_{j+} \bigl( \gamma _{\kappa ^{*},E(\kappa )}(t) \bigr) \\ &\quad \geqslant 0. \end{aligned}$$

In view of $h_{j}(\kappa )\leq 0$, $\beta ^{*}_{j}\geqslant 0, \beta ^{*}_{j}h_{j}(\kappa ^{*})\geqslant (j\in \aleph ) $, and $\zeta ^{*} _{i}= \frac{f_{i}(\kappa ^{*})}{g_{i}(\kappa ^{*})}$ ($i\in P$), then

24 $$ \sum_{i=1}^{p}\alpha ^{*}_{i} \bigl( f_{i}(\kappa )-\zeta _{i}^{*}g_{i}( \kappa ) \bigr)\geqslant 0 \quad \forall y\in Y . $$

Consider that *κ̄* is not a weak efficient solution for (VFP). From $\zeta ^{*}_{i}= \frac{f_{i}(\bar{\kappa })}{g_{i}(\bar{ \kappa })}$ ($i\in P$) and Lemma 3.1, it follows that *κ̄* is not a weak efficient solution for ($\mathrm{VFP}_{\zeta ^{*}} $). Hence, $\tilde{\kappa }\in K $ such that

$$ f_{i}(\tilde{\kappa })-\zeta _{i}^{*}g_{i}( \tilde{\kappa }) < f_{i}(\bar{ \kappa })-\zeta _{i}^{*}g_{i}( \bar{\kappa }) = 0, \quad i\in P. $$

Therefore $\sum_{i=1}^{p}\alpha ^{*}_{i} (f_{i}(\tilde{\kappa })- \zeta _{i}^{*}g_{i}(\tilde{\kappa }) )<0 $. This is a contradiction to inequality (24). The proof of the theorem is completed. □
